# L-Leucine Templated Biomimetic Assembly of SnO_2_ Nanoparticles and Their Lithium Storage Properties

**DOI:** 10.1155/2018/4314561

**Published:** 2018-08-19

**Authors:** Peng Yu, Mili Liu, Haixiong Gong, Fangfang Wu, Zili Yi, Hui Liu

**Affiliations:** ^1^College of Science, Hunan Agricultural University, Changsha 410128, China; ^2^College of Bioscience and Biotechnology, Hunan Agricultural University, Changsha 410128, China

## Abstract

SnO_2_ nanoparticles have been synthesized by a novel route of a sol-gel method assisted with biomimetic assembly using L-leucine as a biotemplate. The microstructure of as-prepared SnO_2_ nanoparticles was characterized by X-ray diffraction (XRD), scanning electron microscopy (SEM), Fourier transform infrared spectra (FT-IR), and Brunner−Emmet−Teller (BET) measurements. The results demonstrated that the growth of SnO_2_ could be regulated by L-leucine at a high calcination temperature. The electrochemical performance of SnO_2_ was also measured as anodes for lithium-ion battery. It is a guidance for the growth regulation of SnO_2_ at high temperature to obtain SnO_2_/C with nanosized SnO_2_ coated by a graphitic carbon.

## 1. Introduction

Lithium-ion batteries (LIBs) are the dominant power supply for portable electronics and also show promising applications for electric vehicles and power storage systems, due to their high specific energy, good cycling performance, high coulombic, and energy efficiency [1, 2]. In accordance with the increasing energy requirement for these industries, LIBs develop toward higher capacity, higher energy, and higher power. Therefore, it is of great significance to explore electrode materials with high capacity for the next generation LIBs. Graphite is the principal commercialized anode for LIBs since invented in 1991, but its explored capacity has been reached the theoretical limit (372 mAh/g) [3–10]. Metal oxides (Co_3_O_4_, Fe_3_O_4_, and SnO_2_) have been studied as anodes to enhance energy density of LIBs, for they can deliver higher capacity than graphite [11–13]. In this regard, SnO_2_ has been considered as an outstanding alternative to graphite because of its high theoretical capacity of 782 mAh/g and moderate lithiation potential (∼0.6 V vs. Li^+^/Li) [8, 14–17]. It is commonly recognized that SnO_2_ experiences a two-step lithiation process, namely,

(1) $$\begin{array}{c} SnO_{2}+4Li^{+}+4e^{-}⟶Sn+2Li_{2}O \end{array}$$

(2) $$\begin{array}{c} Sn+4.4Li^{+}+4.4e^{-}⟶Li_{4.4}Sn \end{array}$$

The reaction shown in (1) is irreversible, which would induce low initial coulombic efficiency (CE) of ~50%. Additionally, SnO_2_ electrode would suffer a large volume variation (~260%) resulted by the reaction of (2) as well as Sn, which would cause crack and collapse. And then, the capacity fading of SnO_2_ is dramatical during cycling. To improve cycling performance for SnO_2_, tremendous investigations have indicated that SnO_2_/C composite anode with nanosized SnO_2_-coated carbon is the most effective strategy [15, 18–20]. In SnO_2_/C electrode, nanosized SnO_2_ could sustain large volume change of Sn during lithiation and delithiation and carbon can buffer the volume change and also maintain the conductivity network for the whole electrode. Therefore, the cycling life of SnO_2_/C would greatly be extended. However, most previous studies presented that SnO_2_ was usually coated by amorphous carbon via the pyrolysis of carbonaceous organic material at 400~500°C, such as glucose. Unfortunately, amorphous carbon with a higher specific surface area might bring large amounts of side reactions with electrolyte, leading to a lower initial CE of SnO_2_. Besides, amorphous carbon usually shows higher average lithiation/delithiation voltage and larger voltage hysteresis, which would contribute little improvement in terms of energy density for LIBs in fact. Now, even since it has been reported that amorphous carbon can be catalytically graphitized at a lower temperature of about 600~700°C [21–23], SnO_2_ particles would grow greatly large at this temperature and experience a rapid capacity fading as lithium-ion anodes. Accordingly, it is of importance to obtain nanosized SnO_2_ and suppress its growth at 600~700°C for the application implementation of SnO_2_/C.

In this work, nanosized SnO_2_ were synthesized by a sol-gel method assisted with biomimetic assembly. Biomimetic synthesis is a novel route to fabricate nanosized inorganic particles with organic templates. Investigations have identified that specific molecular interactions at inorganic-organic interfaces could result in the controlled nucleation and growth of inorganic crystals [24–26]. During the biomimetic assembly process, the organic template could promote self-assembly, recognize the reactant substrate, guide the nucleation, and limit the growth of inorganic particles by utilizing biological adsorption, hydrogen bond, van der Waals force, and so on. Considering that L-leucine could regulate the synthesis and the growth of organic particles and even enzymes, while no researches related to the regulation of inorganic materials by L-leucine could be found [27], we would like to control SnO_2_ nucleation and growth by biomimetic assembly using L-leucine as a biomimetic template here. The regulated mechanism of SnO_2_ synthesis and growth is studied for the first time in this work. Moreover, the electrochemical performance of as-prepared SnO_2_ was also measured as lithium-ion anodes.

## 2. Experimental

### 2.1. Preparation of Materials

SnO_2_ nanoparticles were synthesized by a sol-gel method assisted with biomimetic assembly using leucine as a biotemplate. Firstly, 9% of dilute aqua ammonia was dripped slowly into SnCl_4_ (0.4 M/40 ml) including 0.001 mol L-leucine with continuous stirring in a water bath under 65°C. Secondly, the pH of the as-produced solution was adjusted to 3 and then kept stirring for 1 h to get white sol. Finally, the resulting sol was centrifuged, washed with ethanol and deionized water three times after keeping stand under 80°C for 24 h, respectively, and then dried at 80°C. The resultant was heated to 450°C, 550°C, and 650°C at a ramp rate of 10°C·min^−1^ in the air for 4 h to get SnO_2_, named as L-SnO_2_-450°C, L-SnO_2_-550°C, and L-SnO_2_-650°C, respectively. For comparison, SnO_2_ nanoparticles were also prepared using the same procedure without the L-leucine biotemplate, named as SnO_2_-450°C, SnO_2_-550°C, and SnO_2_-650°C.

### 2.2. Material Characterization

The microstructure of SnO_2_ particles was carried out by a Shimadzu X-ray 6000 diffractometer (XRD) with CuK*_α_* radiation at 40 kV, 30 mA and a Quanta 250 FEG scanning electron microscope (SEM). Fourier transform infrared (FTIR) spectra were recorded using a Bruker Alpha spectrometer. Brunner−Emmet−Teller (BET) measurements were recorded using a QUADRASORB SI analyzer.

### 2.3. Electrochemical Measurements

The electrode slurry was prepared by mixing 90 wt% active material, 2 wt% super-p, and 8 wt% carboxymethyl cellulose (CMC). And then, the slurry was spread on Cu foil and dried at 120°C for 1 h. The electrochemical measurements of the electrodes were tested using CR2032 coin cells assembled with Li foil as the counter and reference electrodes in an argon-filled glove box. The electrodes were separated by two layers of a Celgard separator. The electrolyte was 1 M LiPF_6_ dissolved in a mixture of ethylene carbonate, ethyl methyl carbonate, and dimethyl carbonate (EC : EMC : DMC = 1 : 1 : 1 in volume).

Cycle test was conducted with a system of LAND CT2001A from 0.005 to 2.5 V at a 0.1 C rate and trickle discharged at 0.005 V to a C/40 rate in the first three cycles. For subsequent cycles, cells were cycled from 0.005 to 2.5 V at a 0.2 C rate and trickle discharged at 0.005 V to a C/20 rate. All electrochemical tests were carried out at ambient temperature.

## 3. Results and Discussion

**Figure 1:**
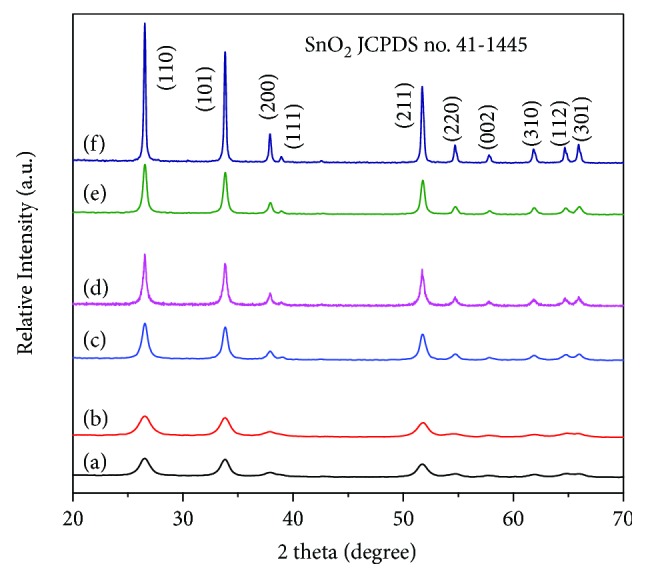
XRD patterns of as-prepared SnO_2_ nanoparticles divided into three groups of (a) L-SnO_2_-450°C and (b) SnO_2_-450°C, (c) L-SnO_2_-550°C and (d) SnO_2_-550°C, and (e) L-SnO_2_-650°C and (f) SnO_2_-650°C.

Figure 1 compares the XRD patterns of as-prepared SnO_2_ nanoparticles divided into three groups of (a) L-SnO_2_-450°C and (b) SnO_2_-450°C, (c) L-SnO_2_-550°C and (d) SnO_2_-550°C, and (e) L-SnO_2_-650°C and (f) SnO_2_-650°C. All patterns show obvious diffraction peaks at 2*θ* of 26.61°, 33.89°, 37.95°, 51.78°, and 65.94°, corresponding to (110), (101), (200), (211), and (301) planes of rutile SnO_2_ (JCDF no. 41-1445), respectively. No peak corresponding to crystallographic impurities was observed, indicating the high purity of SnO_2_. As the increase of calcination temperature from 450°C to 650°C, the diffraction peaks become sharp and the full width at half maximum (FWHM) narrows significantly for both L-SnO_2_ and SnO_2_, which is an indication that the grain size of SnO_2_ increases with an increase of calcination temperature. What is more, it could be observed that the grain size of SnO_2_ is finer in L-SnO_2_ than that of SnO_2_ prepared without leucine templates under the same temperature. Moreover, such distinctions in SnO_2_ grain size get more evident as the calcination temperature increases.

Compared Figure 1(a) and (b), the FWHM of the SnO_2_ diffraction peak is very close for L-SnO_2_-450°C and SnO_2_-450°C, while it is obviously wide in L-SnO_2_-550°C than SnO_2_-550°C as shown in Figure 1(c) and (d). Especially in Figure 1(e) and (f), the diffraction peaks become much sharper in SnO_2_-650°C than L-SnO_2_-650°C. Based on peak profile analysis using a Voigt function, it is confirmed that the grain size of SnO_2_ is calculated as 11.2, 16.7, and 20.5 nm for L-SnO_2_-450°C, L-SnO_2_-550°C, and L-SnO_2_-650°C, respectively, while 11.0, 23.0, and 39.2 nm for SnO_2_-450°C, SnO_2_-550°C, and SnO_2_-650°C, respectively. It could be concluded that the SnO_2_ growth can be suppressed when synthesized by a sol-gel method assisted with biomimetic assembly using leucine as a biotemplate.

**Figure 2:**
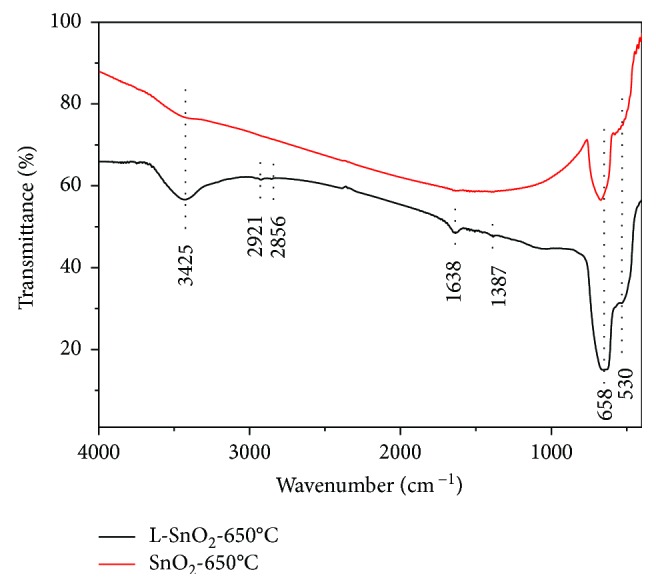
FT-IR spectrum of synthesized L-SnO_2_-650°C and SnO_2_-650°C.

Figure 2 shows the FT-IR spectra of L-SnO_2_-650°C and SnO_2_-650°C. The main peak of L-SnO_2_-650°C and SnO_2_-650°C is at the same position of 658 cm^−1^ attributed to the Sn-O-Sn asymmetric stretching mode of surface bridging oxide. It confirms SnO_2_ formation in L-SnO_2_-650°C and SnO_2_-650°C. The weak peak is around 3425 cm^−1^ in SnO_2_-650°C corresponding to the stretching vibration of O-H bond, which may be due to the presence of water molecule on the surface of SnO_2_ nanoparticles and the stretching vibrations of Sn-OH groups [28, 29]. However, the peak of 530 cm^−1^ assigned to Sn-O vibration of Sn-OH group is weak in SnO_2_-650°C. Therefore, it could be considered that the peak around 3425 cm^−1^ is mainly due to the vibration of absorbed water molecules. A stronger peak of 3425 cm^−1^, together with 1387 cm^−1^ of -CH_3_ and 1638 cm^−1^ of -NH_2_ in L-SnO_2_-650°C, is an indication of L-leucine residuum bonding with SnO_2_, though Sn(OH)_4_ precursor was washed by ethanol and deionized water before calcination.

**Figure 3:**
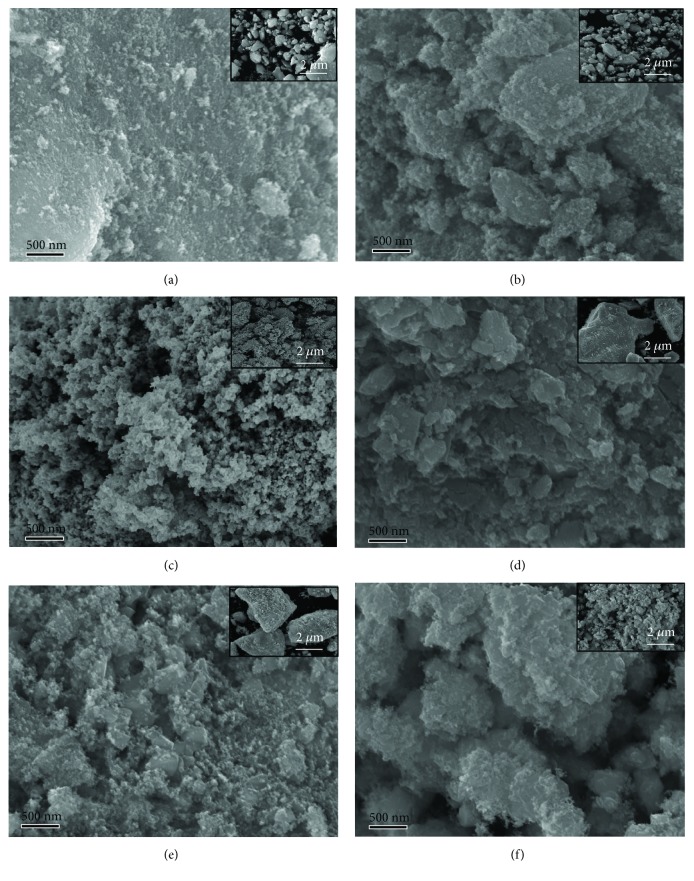
SEM images of (a) L-SnO_2_-450°C, (b) L-SnO_2_-550°C, (c) L-SnO_2_-650°C, (d) SnO_2_-450°C, (e) SnO_2_-550°C, and (f) SnO_2_-650°C.

It is predicted that the growth of SnO_2_ particles in L-SnO_2_ gets suppressed for its direction and rate of interfacial migration between individual grains is regulated by L-leucine. This should maintain a block structure accompanied with wrinkled morphology and retain a smooth and dense surface structure for L-SnO_2_, while a large number of scattered particles and flakes increase to the surface of the control SnO_2_ group. When calcined at 650°C as shown in Figures 3(c) and 3(f), the block structure of L-SnO_2_-650°C and SnO_2_-650°C has damaged at a certain degree, with SnO_2_ nanoparticles reuniting on the various surfaces and edges along the block. It should be noted that L-SnO_2_-650°C shows highly porous foam-like morphology.

**Figure 4:**
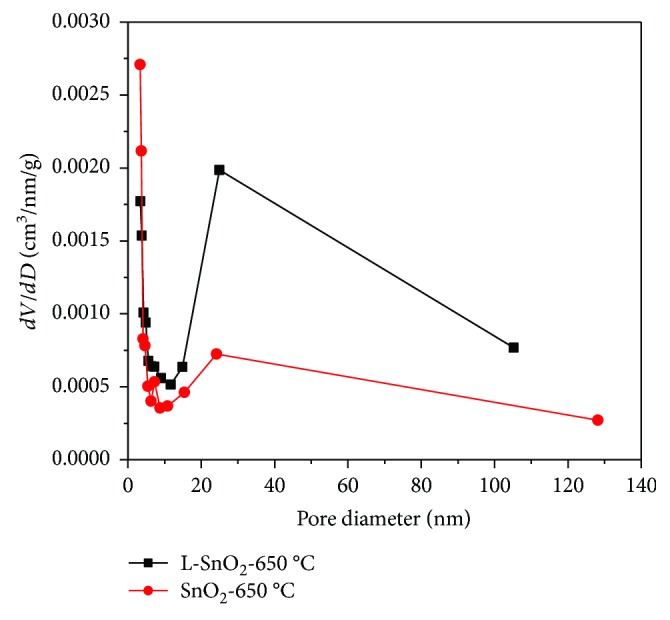
Pore size distribution of L-SnO_2_-650°C and SnO_2_-650°C.

To observe the porous structure of L-SnO_2_-650°C, Figure 4 shows the pore size distribution curves for L-SnO_2_-650°C and SnO_2_-650°C and Table 1 shows the surface area, pore volume, and pore diameter of L-SnO_2_-650°C and SnO_2_-650°C. Compared with SnO_2_-650°C, L-SnO_2_-650°C possesses more mesopores of about 30 nm, the pore volume of 0.15 cm^3^/g is much bigger than 0.07 cm^3^/g, and the pore diameter of 24.9 nm is eight times than 3.3 nm. Therefore, the surface area of L-SnO_2_-650°C is nearly twice larger than that of SnO_2_-650°C.

**Figure 5:**
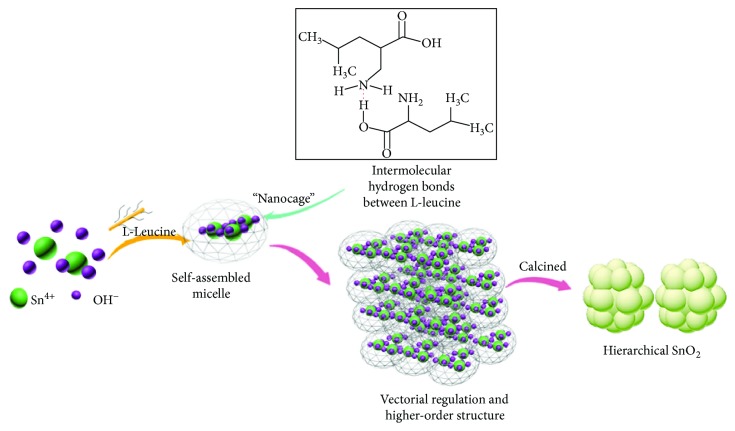
The formation of “nanocage” and intermolecular hydrogen bonds between L-leucines.

**Figure 6:**
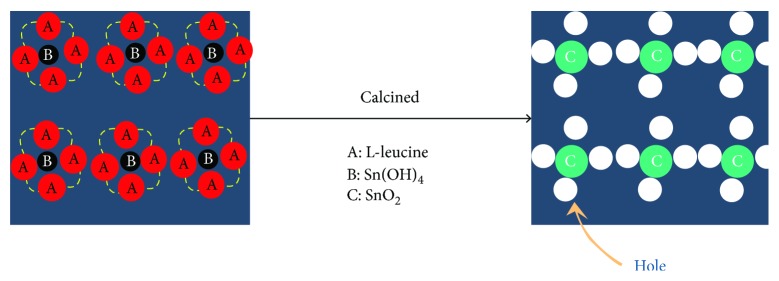
Schematic for the foam-like morphology of L-SnO_2_-650°C.

Based on above microstructure characterization, the mechanism of SnO_2_ synthesis by biomimetic assembly could be schematic in Figures 5 and 6. First, -NH_2_ of L-leucine accelerates the self-assembled process of Sn^4+^ and OH^−^ to form Sn(OH)_4_. And then, Sn(OH)_4_ could be recognized and integrated with L-leucine. In addition, -COOH of L-leucine, an electron-withdrawing group, easily form a hydrogen bond with -NH_2_ of the next L-leucine molecule. Therefore, a “nanocage” with L-leucine molecules enclosed with Sn(OH)_4_ would be created by the intermolecular hydrogen bonding of L-leucine. Lastly, a high-order nanocage group would be formed. When heating Sn(OH)_4_, SnO_2_ nucleates in the “nanocage” and its growth would be restricted by the “nanocage.” Therefore, fine and high-order layered SnO_2_ particles could be obtained by biomimetic assembly using the L-leucine template.  Figure 6 illustrates the formation mechanism of the porous foam-like surface for L-SnO_2_. L-Leucine has integrated with Sn(OH)_4_ to form a “nanocage” group, which would not be washed. When calcined at a high temperature, L-leucine would decompose into gaseous product such CO_2_, NH_2_, and CO as reported in [30]. Then, the escapement of these gases and the pyrolysis removal of the L-leucine template would leave holes around SnO_2_ and promote the formation of porous morphology.

**Figure 7:**
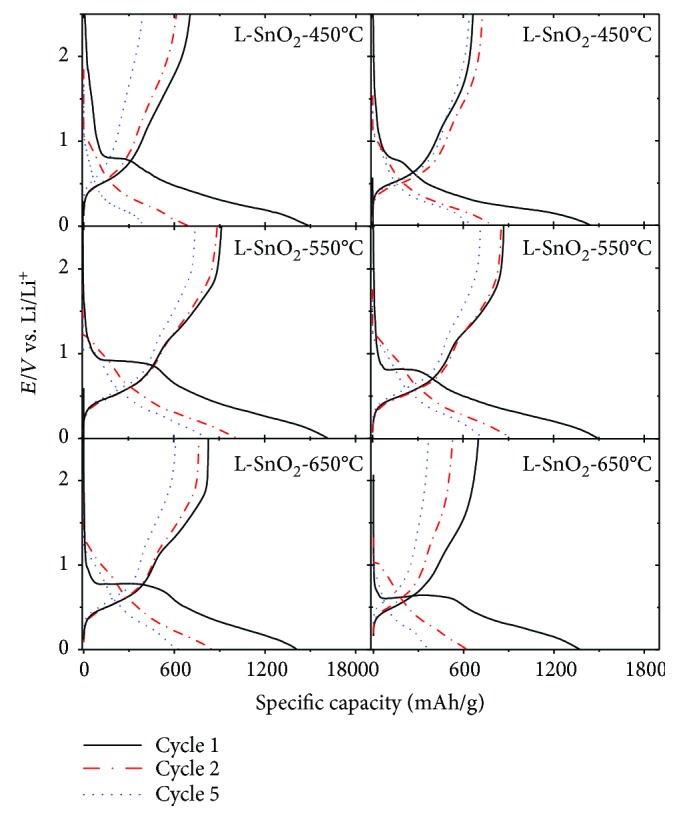
The 1st, 2nd, and 5th voltage-capacity profiles of as-prepared SnO_2_ electrodes.

Figure 7 shows voltage-capacity curves of the 1st, 2nd, and 5th cycles for as-prepared SnO_2_ electrodes cycled versus lithium metal from 0.005 to 2.5 V. All the voltage curves are characteristic of SnO_2_ in appearance, having an initial discharge sloping above 1.0 V, a flat plateau about 0.8 V, and a sloping plateau during the subsequent lithiation and delithiation process. A large irreversible capacity at above 1.0 V during the first discharge could also be observed, corresponding to the irreversible reaction between SnO_2_ and Li (1). The flat plateau about 0.8 V is consistent with the reaction between Li^+^ and Sn. The L-SnO_2_-450°C, L-SnO_2_-550°C, and L-SnO_2_-650°C deliver the initial discharge capacity of 1488.3 mAh/g, 1616.1 mAh/g, and 1408.9 mAh/g, respectively, which is higher than 1441.3 mAh/g, 1491.7 mAh/g, and 1370.0 mAh/g of SnO_2_-450°C, SnO_2_-550°C, and SnO_2_-650°C, respectively. And the initial charge capacity of L-SnO_2_ is also higher than the latter, with 704.0 mAh/g, 914.9 mAh/g, and 824.8 mAh/g for L-SnO_2_-450°C, L-SnO_2_-550°C, and L-SnO_2_-650°C, respectively, while 655.1 mAh/g, 869.7 mAh/g, and 698.3 mAh/g for SnO_2_-450°C, SnO_2_-550°C, and SnO_2_-650°C, respectively. Additionally, the compacted density is 3.63 g/cm^3^, 3.74 g/cm^3^, and 3.38 g/cm^3^ for L-SnO_2_-450°C, L-SnO_2_-550°C, and L-SnO_2_-650°C, respectively. So, the corresponding reversible volumetric capacity is 982.9 mAh/cm^3^, 1316.0 mAh/cm^3^, and 1072.2 mAh/cm^3^, respectively, which is higher than commercial graphite of 720 mAh/cm^3^. Moreover, the flat plateau of L-SnO_2_ is a little higher than single SnO_2_ calcined at the same temperature. This should be related to smaller SnO_2_ in L-SnO_2_, so they could provide more passageways for Li^+^ diffusion, deliver more capacity, and react with Li^+^ easily to form Li*_z_*Sn. Additionally, L-SnO_2_-450°C and L-SnO_2_-550°C perform a coulombic efficiency of 47.3% and 56.6%, respectively, which is equal to 45.5% and 58.3% for SnO_2_-450°C and SnO_2_-550°C, respectively. This would be concluded that the functional groups in the surface of SnO_2_ have no irreversible capacity contribution.

**Figure 8:**
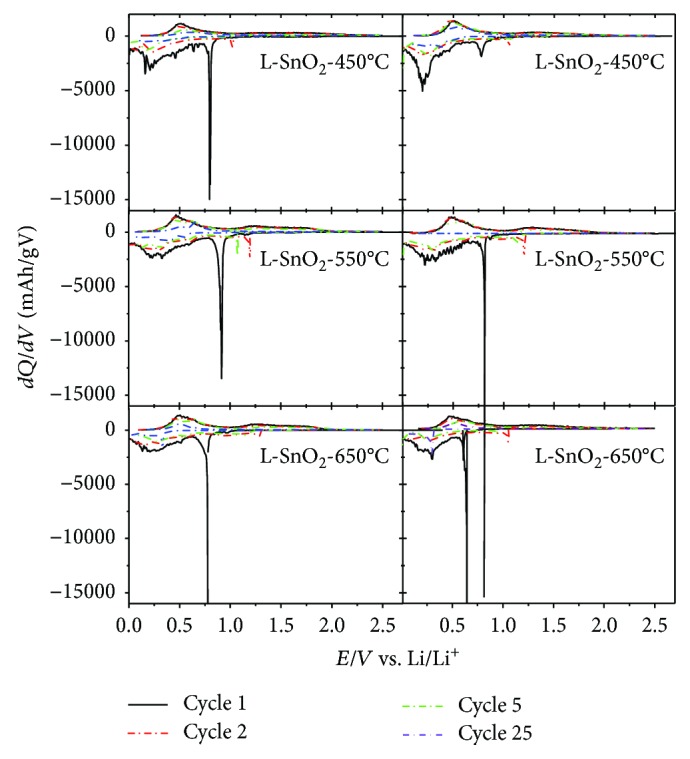
Differential capacity vs. potential curves of the 1st, 2nd, 5th, and 25th cycles for L-SnO_2_ electrodes and the control group of SnO_2_ electrodes.

Figure 8 shows the differential capacity versus potential curves for the 1st, 2nd, 5th, and 25th cycles for as-prepared SnO_2_ electrodes. The differential capacity refers to the calculated value of two adjacent points on the voltage-time curve (*V*_(*n*)_, *V*_(*n* + 1)_, *t*_(*n*)_, *t*_(*n* + 1)_), and the known charge, discharge current *I*, and the value of the active material mass *m* in the electrode according to *dQ*/*dV* = (*I*[*t*(*n* + 1) − *t*(*n*)])/(*m*[*V*(*n* + 1) − *V*(*n*)]). Researchers have testified that the reversibility of lithiation and delithiation of SnO_2_ should be good while the differential capacity curve is broad. The peaks on the differential capacity curve correspond to the platform of the voltage-capacity curve. The change of the area is enclosed by the curve which reflects the attenuation degree of the capacity; the area changes larger, and capacity attenuates faster. As shown in Figure 8, L-SnO_2_-450°C, L-SnO_2_-550°C, and L-SnO_2_-650°C show a sharp peak around 0.80 V, 0.90 V, and 0.77 V during the first discharge, respectively, which is higher than 0.78 V, 0.81 V, and 0.65 V of SnO_2_-450°C, SnO_2_-550°C, and SnO_2_-650°C, respectively. This phenomenon would also exist during the 2nd and 5th discharges, with lithiation peaks at 0.20 V, 0.33 V, and 0.34 V for L-SnO_2_-450°C, L-SnO_2_-550°C, and L-SnO_2_-650°C, respectively, while 0.20 V, 0.27 V, and 0.30 V for SnO_2_-450°C, SnO_2_-550°C, and SnO_2_-650°C, respectively. This suggests that smaller SnO_2_ is easy to alloy with Li^+^. No sharp peak could be observed after the first charge curves, which manifests that no 2-phase district exists. The reductive electric potentials around 0.3 V are also a featured platform of Sn corresponding to Sn and Li formed an alloy. The differential capacity curve of the 25th discharge process was basically a smooth state, and it proves that there is some irreversible oxidation-reduction reaction happened. This phenomenon was associated with electrolyte consumption of SEI on the fresh surface of electrodes. Sn would reunite and easily collapse in charge and discharge processes. Thus, SEI should reform on the exposed fresh surface of Sn. As for the smoothness of *dQ*-*dV* curves, peak of L-SnO_2_ is broader than single SnO_2_, suggesting that L-SnO_2_ have preferable transmission property for lithiation and delithiation. Therefore, the reason that the capacity of L-SnO_2_-450°C was less than SnO_2_-450°C is due to the adsorption of functional groups on the surface of the material, thus blocked the passage of the electrons and led to a decrease in the lithium storage capacity of the material. As for L-SnO_2_-550°C and L-SnO_2_-650°C, their curves were also smoother as well as indicated advantageous performance than SnO_2_-550°C and SnO_2_-650°C.

**Figure 9:**
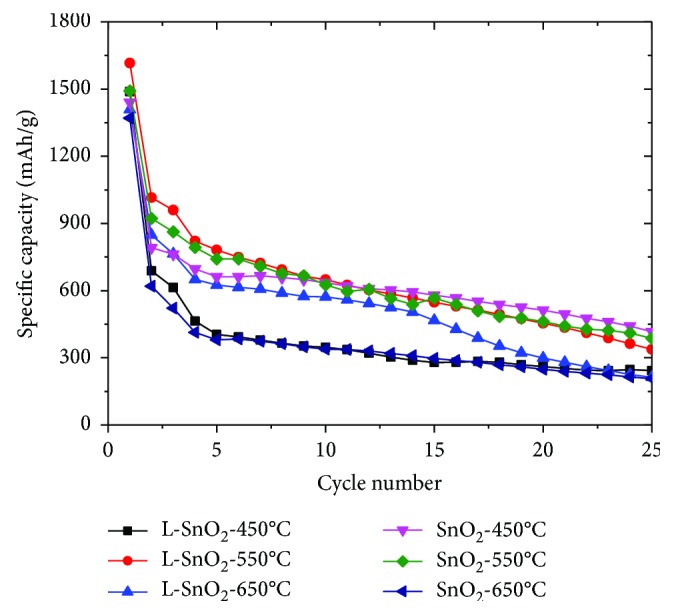
Cyclic performance of L-SnO_2_ electrodes and the control group of SnO_2_ electrodes.

Figure 9 shows the cycling performance for L-SnO_2_ electrodes and the control group of SnO_2_ electrodes. Compared L-SnO_2_-450°C with SnO_2_-450°C, the capacity retention of L-SnO_2_-450°C is inferior to that of SnO_2_-450°C. It should be mainly related to the larger specific surface area for L-SnO_2_-450°C while the particle size of SnO_2_ is close. The cyclic performance of L-SnO_2_-550°C and L-SnO_2_-650°C is much better than the SnO_2_-550°C and SnO_2_-650°C, because the particle size of SnO_2_ is much smaller in the former, which would experience less inner stress. Moreover, the porous structure of L-SnO_2_ can provide more channels and placeholders for the embedding and deembedding of ions. This was helpful to reduce SnO_2_ crushing and improve the stability of electrode materials. Even so, all SnO_2_ electrodes do not show expected excellent cyclability as well as those reported previously. However, it is believed that nanosized SnO_2_ coated by graphitic carbon at 600~700°C would perform better cyclability, which would be further studied.

## 4. Conclusion

SnO_2_ nanoparticles have been prepared by biomimetic synthesis combined with a sol-gel method using L-leucine as a biotemplate for the first time. L-Leucine could form a “nanocage” by its intermolecular hydrogen bond and accelerate the assembly of Sn^4+^ and OH^−^ in the nanocage during the preparation process. Therefore, SnO_2_ growth could be regulated at a high temperature calcination of 650°C. As-prepared L-SnO_2_ show a block and porous structure. As anodes for lithium-ion battery, L-SnO_2_ perform better electrochemical performance than SnO_2_. This should give a promising route to produce enhanced SnO_2_/C electrodes with nanosized SnO_2_ coated by graphitic carbon at high temperature for lithium-ion batteries.

## Figures and Tables

**Table 1:** The surface area, pore volume, and pore diameter of L-SnO_2_-650°C and SnO_2_-650°C.

Sample	Surface area (m^2^/g)	Pore volume (cm^3^/g)	Pore diameter (nm)
L-SnO_2_-650°C	14.7	0.15	24.9
SnO_2_-650°C	7.8	0.07	3.3

## Data Availability

All data generated or analyzed during this study are included in this published article (and its supplementary information files).
